# Early diagnostic value of serum S100 calcium-binding protein A12, serum amyloid A protein, and neutrophil-to-lymphocyte ratio in patients with acute empyema

**DOI:** 10.3389/fcimb.2026.1740584

**Published:** 2026-07-09

**Authors:** Xiao-Lian Zhou, Dong-Qiong Li, Duo Li

**Affiliations:** 1 Department of Pulmonary and Critical Care Medicine, West China School of Medicine, Sichuan University, Sichuan University Affiliated Chengdu Second People's Hospital, Chengdu Second People's Hospital, Chengdu, Sichuan, China; 2 Department of Pulmonary and Critical Care Medicine, Xichang People's Hospital, Xichang, Sichuan, China; 3 Department of Pulmonary and Critical Care Medicine, Southwest Medical University, Luzhou, Sichuan, China

**Keywords:** acute empyema, diagnostic value, NLR, S100A12, SAA

## Abstract

**Background:**

Acute empyema refers to suppurative inflammation of the pleural cavity secondary to pulmonary infection. Prompt diagnosis and intervention are required upon identification to prevent progression to chronic empyema; hence, clinically effective biomarkers are needed to predict the development of acute empyema.

**Objective:**

To investigate the early diagnostic value of four serum markers, namely, S100 calcium-binding protein A12 (S100A12), serum amyloid A (SAA), C-reactive protein (CRP), and neutrophil-to-lymphocyte ratio (NLR), for predicting progression to acute empyema in patients with early-stage pneumonia.

**Methods:**

A retrospective analysis was performed on 60 patients diagnosed with community-acquired pneumonia complicated with acute empyema admitted to Xichang People’s Hospital from January 2021 to June 2022. Another 60 patients with community-acquired pneumonia without empyema treated during the same period were enrolled as controls. Univariate analysis was used to compare differences in general clinical data, clinical manifestations, biochemical indicators, and treatment conditions between the two groups. Receiver operating characteristic (ROC) curves were adopted to evaluate differences in sensitivity and specificity of single and combined detection of S100A12, SAA, and NLR for early identification of acute empyema.

**Results:**

Levels of S100A12, SAA, CRP, and NLR were significantly higher in patients with acute empyema than in those with community-acquired pneumonia (all *P* < 0.05), whereas serum albumin was markedly lower in the empyema group (*P* < 0.05). ROC curve analysis was performed to assess the diagnostic efficacy of individual and combined S100A12, SAA, and NLR for predicting acute empyema. The combined panel of S100A12+SAA+NLR yielded a sensitivity of 76.7% and a specificity of 96.7%, with an area under the ROC curve (AUROC) of 0.938 (95% CI: 0.900–0.977, *P* < 0.0001). The AUROC of the combined panel was significantly superior to those of single S100A12 (AUROC = 0.803, 95% CI: 0.722–0.884, *P* < 0.0001), SAA (AUROC = 0.908, 95% CI: 0.860–0.957, *P* < 0.0001), and NLR (AUROC = 0.694, 95% CI: 0.599–0.788, *P* = 0.0003), with statistically significant differences (*P* < 0.05).

**Conclusion:**

Acute empyema is mostly secondary to pulmonary infection, which is closely associated with host immunity, bacterial virulence, and invasiveness. Clinicians need reliable predictive biomarkers for early identification and prevention of acute empyema progression. Our study reveals that the combined panel of S100A12, SAA, and NLR achieves favorable sensitivity and specificity in predicting acute empyema, exhibiting superior diagnostic performance for early detection and promising clinical application prospects.

## 1 Introduction

Community-acquired pneumonia (CAP) is a common type of pulmonary infection. Severe pulmonary infection or delayed treatment may trigger multiple pulmonary complications including acute empyema, also known as community-acquired suppurative pleuritis. Previous studies have demonstrated that up to half of patients with severe pulmonary infection progress to complicated parapneumonic effusion (Lisboa et al., 2011). Empyema is a high-mortality suppurative disease caused by bacterial invasion of the pleural cavity secondary to pulmonary infection. Epidemiological evidence indicates a rising overall incidence attributable to multiple interacting contributors: aging population, growing high-risk populations with chronic comorbidities, expanded clinical use of immunosuppressive agents, spontaneous variation in pathogenic bacteria, and a male-to-female predominance of approximately 2.3:1 (Bedawi et al., 2023). In the UK and the United States, over 65,000 patients suffer from pleural infection annually. Despite more than two millennia of clinical experience alongside advances in modern healthcare and antibiotic therapy, pleural infection remains a prominent contributor to morbidity and mortality across both developed and developing nations in the 21st century (Lisboa et al., 2011). Among CAP patients, acute empyema represents one of the leading causes of treatment failure and poor prognosis, frequently prolonging therapeutic courses and hospital stay (Cillóniz et al., 2012). Accordingly, there is an urgent clinical demand for convenient, rapid predictive biomarkers with robust diagnostic efficacy to enable early identification and diagnosis of acute empyema.

## 2 Methods

### 2.1 Subjects and methods

#### 2.1.1 Study subjects

This retrospective study enrolled 60 inpatients diagnosed with community-acquired pneumonia complicated by acute empyema who met the inclusion and exclusion criteria and were admitted to the Department of Respiratory and Critical Care Medicine of our hospital between January 2021 and June 2022 as the case group. Meanwhile, another 60 patients with community-acquired pneumonia without empyema were randomly selected from contemporary hospitalized cases to form a matched control group. A total of 92 men and 28 women were included, aged 18 to 86 years with a mean age of (54.57 ± 16.680) years. This retrospective study was exempted from written informed consent and approved by the Institutional Research Ethics Committee of Xichang People’s Hospital.

### 2.2 Inclusion and exclusion criteria

#### 2.2.1 Inclusion criteria

Patients in the control group were diagnosed in accordance with the Chinese Guidelines for the Diagnosis and Treatment of Adult Community-Acquired Pneumonia (2016 Edition) (Qu and Cao, 2016). In addition to meeting the above CAP criteria, subjects in the case group should satisfy the diagnostic criteria for acute empyema as follows: imaging findings suggestive of pleural effusion accompanied by empyema-related manifestations such as pleural loculation and pleural thickening; pus-like gross appearance of pleural fluid obtained via thoracentesis; and markedly elevated white blood cell count predominantly consisting of neutrophils on routine pleural fluid testing. Patients meeting any two of these three items together with infectious clinical manifestations can be clinically diagnosed with empyema.

#### 2.2.2 Exclusion criteria

The exclusion criteria were as follows: 1) infections caused by tuberculosis, fungi, or opportunistic pathogens; 2) primary empyema; 3) immunocompromised status, including HIV infection, hematological malignancies, solid organ or bone marrow transplantation, and ongoing immunosuppressive treatment; 4) hospital-acquired pneumonia; 5) pleural effusion resulting from other etiologies such as tuberculosis or malignant tumors; and 6) patients with incomplete clinical data.

### 2.3 Methods

All patients received standardized treatment after admission. Routine laboratory and imaging examinations, as well as serum measurements of S100 calcium-binding protein A12 (S100A12), serum amyloid A (SAA), and C-reactive protein (CRP), were performed within 24–48 h after the onset of fever, cough, chest pain, and other relevant symptoms. Serum S100A12 was detected strictly following the instructions of the S100A12 assay kit and the corresponding equipment operating specifications.

The experimental instruments and detection method used were as follows:

Microplate reader: Tecan Infinite F50;
Microplate washer: automatic DKW-series microplate washer (Model DKW-330);
Centrifuge: high-speed refrigerated centrifuge (Model CenLee16R);
Incubator: electric thermostatic incubator (Model DH124D);
Detection method: enzyme-linked immunosorbent assay (ELISA).

#### 2.3.1 Diagnostic procedures

Diagnosis of acute empyema: Once pleural effusion is identified on chest radiograph or chest computed tomography (CT), approximately 20 mL of pleural fluid is collected via thoracentesis under aseptic conditions and processed in accordance with standard protocols. After macroscopic assessment of pleural fluid (including color, odor, and turbidity), 5 mL of fluid is inoculated into an aerobic culture bottle and another 5 mL into an anaerobic culture bottle for routine microbial culture. Subsequent bacterial identification and antimicrobial susceptibility testing are conducted for isolated pathogens, alongside biochemical analysis of pleural fluid consisting of pus cell count, pH value, lactate dehydrogenase, and total protein quantification (Falguera et al., 2011). Differential diagnosis of parapneumonic effusion is required to distinguish from malignant pleural effusion, tuberculous pleural effusion, and heart failure-related pleural effusion (Li et al., 2019), which is comprehensively determined based on medical history, clinical manifestations, physicochemical properties of pleural fluid, differential cell count, pleural inflammatory biomarkers, and imaging findings. Pus culture from thoracentesis specimens enables the identification of specific pathogenic bacteria; nevertheless, bacteriological confirmation via pus culture remains challenging for empyema patients. Failed bacterial culture results can be attributed to multiple factors: intrinsic difficulty in cultivating anaerobic bacteria, prior antibiotic administration before specimen collection, accidental aspiration of sterile inflammatory adjacent cavity fluid, and low sensitivity of conventional culture techniques (Walters, 2011; Pearce et al., 2025).

#### 2.3.2 Relevant definitions

Timely and effective drainage: Timely drainage is defined as closed thoracic drainage initiated within 24 h after confirmed diagnosis of acute empyema. Effective drainage is confirmed by unobstructed drainage tube, alleviated infectious symptoms, decreased inflammatory markers, and remarkable absorption of pleural effusion on imaging. According to the ERS/ESTS guidelines, persistent pleural drainage is an essential component of effective management for acute empyema (Li et al., 2024).

Nutritional status assessment: Nutritional status was evaluated using the Mini-Nutritional Assessment (MNA) questionnaire developed by Guigoz et al. (1996). The nutritional screening contains six items (see **Supplementary Material 1** for details), with a total MNA score ranging from 0 to 14 points. Patients with a total score ≥12 points are classified as well-nourished, scores of 8–11 points indicate risk of malnutrition, and a total score <7 points confirms definite malnutrition.

### 2.4 Observation indicators

A retrospective case–control study was conducted. Patients diagnosed with acute empyema were assigned to the case group, while those with community-acquired pneumonia served as the control group. Clinical data of the two groups were collected and analyzed, including 1) baseline characteristics: gender, age, and nutritional status; 2) past medical history: diabetes mellitus, chronic obstructive pulmonary disease (COPD), and chronic alcohol abuse; 3) clinical manifestations: pleural-related symptoms and purulent sputum production; 4) laboratory parameters: serum S100A12, SAA, CRP, serum albumin, and neutrophil-to-lymphocyte ratio (NLR); and 5) treatment-related information: regimens of antibiotic administration and pleural fluid drainage. Differences in the above clinical variables were compared between the two groups to identify effective laboratory markers for early detection and diagnosis of acute empyema.

### 2.5 Statistical analysis

Statistical analyses and database construction were performed using SPSS 22.0 software. Enumeration data were expressed as frequencies and percentages and compared via the chi-square test for univariate analysis. Measurement data were presented as mean ± standard deviation (SD), and intergroup differences were compared using independent samples *t*-test. Receiver operating characteristic (ROC) curves were plotted to calculate the sensitivity, specificity, and area under the curve (AUC) of each biomarker, and pairwise comparisons of AUC values were conducted accordingly. A two-tailed *P <*0.05 was considered statistically significant.

## 3 Results

### 3.1 Comparison of clinical characteristics between the two groups

No statistically significant intergroup differences were observed with respect to age, gender, prevalence of chronic obstructive pulmonary disease, presence of pleural symptoms, and duration of antibiotic therapy prior to pleural fluid sampling (*P* > 0.05). In contrast, significant differences were found regarding nutritional status; diabetes mellitus; alcohol abuse; purulent sputum production; serum levels of S100A12, SAA, CRP, serum albumin, and NLR; and pleural effusion drainage status (all *P* < 0.05), indicating that these variables may constitute risk factors for the development of acute empyema. Specifically, the empyema group exhibited significantly higher S100A12, SAA, CRP, and NLR levels and substantially lower serum albumin concentrations compared with the community-acquired pneumonia group (*P* < 0.05), as summarized in **Table 1**.

**Table 1:** Comparison of clinical characteristics between the two groups.

Characteristics	Factors	Acute pyothorax group (*n* = 60)	Pneumonia group (*n* = 60)	T/χ2	*P*
Gender (M/F)		50/10	42/18	2.981	0.130
Age (years)	>60 ≤60	29 31	22 38	1.671	0.268
Nutritional status (good/moderate/bad)		30/14/16	48/6/6	11.899	0.003
Diabetes (yes/no)		19/41	7/53	7.070	0.014
COPD (yes/no)		15/45	12/48	0.430	0.662
Alcoholism (yes/no)		28/32	8/52	15.873	<0.001
Pleural symptoms (yes/no)		45/15	37/23	2.465	0.169
Cough with pus (yes/no)		43/17	26/34	9.855	0.003
Duration of antibiotic use	<2 weeks ≥2 weeks	30 30	39 21	2.762	0.139
Drainage of pleural fluid (adequate/inadequate)		31/29	47/13	9.377	0.004
S100A12 (ng/mL)		27.8028 ± 23.78856	12.5803 ± 12.48227	4.389	<0.001
SAA (mg/L)		163.0672 ± 87.03827	44.8128 ± 38.84148	9.611	<0.001
CRP (mg/L)		125.5625 ± 65.36309	67.3168 ± 57.69371	5.175	<0.001
Serum albumin (g/L)		31.6127 ± 4.76310	38.3783 ± 5.04804	−7.551	<0.001
NLR		14.9583 ± 11.78119	8.3828 ± 6.77539	3.748	<0.001

### 3.2 Differences in inflammatory biomarkers among subgroups with distinct prognoses in 120 pneumonia patients

All 120 adult patients with CAP were followed up. Taking 30-day prognostic outcome as the dependent variable and S100A12, SAA, CRP, and NLR as independent variables, respectively, univariate and multivariate logistic regression analyses were performed. Multivariate analysis identified S100A12 (OR = 1.050, 95% CI: 1.012–1.089), SAA (OR = 1.103, 95% CI: 1.052–1.157), and NLR (OR = 1.090, 95% CI: 1.008–1.180) as independent risk factors for progression to empyema within 30 days in adult CAP patients (all *P*<0.05, **Table 2**). By contrast, CRP yielded an OR of 0.949 (95% CI: 0.921–0.978), indicating that elevated CRP was associated with a reduced risk of empyema transformation from pneumonia, with statistically significant differences (*P* < 0.05).

**Table 2:** Multivariate logistic regression analysis for 30-day prognosis in adult patients with CAP.

Factors	*B*	S.E.	Wald	OR	95%CI	*P*
S100A12 (ng/mL)	0.049	0.019	6.681	1.050	1.012–1.089	0.010
SAA (mg/L)	0.098	0.024	16.650	1.103	1.052–1.157	<0.001
CRP (mg/L)	−0.052	0.015	11.564	0.949	0.921–0.978	0.001
NLR	0.086	0.040	4.624	1.090	1.008–1.180	0.032

### 3.3 ROC curve analysis of S100A12, SAA, and NLR for predicting acute empyema

**Figure 1:**
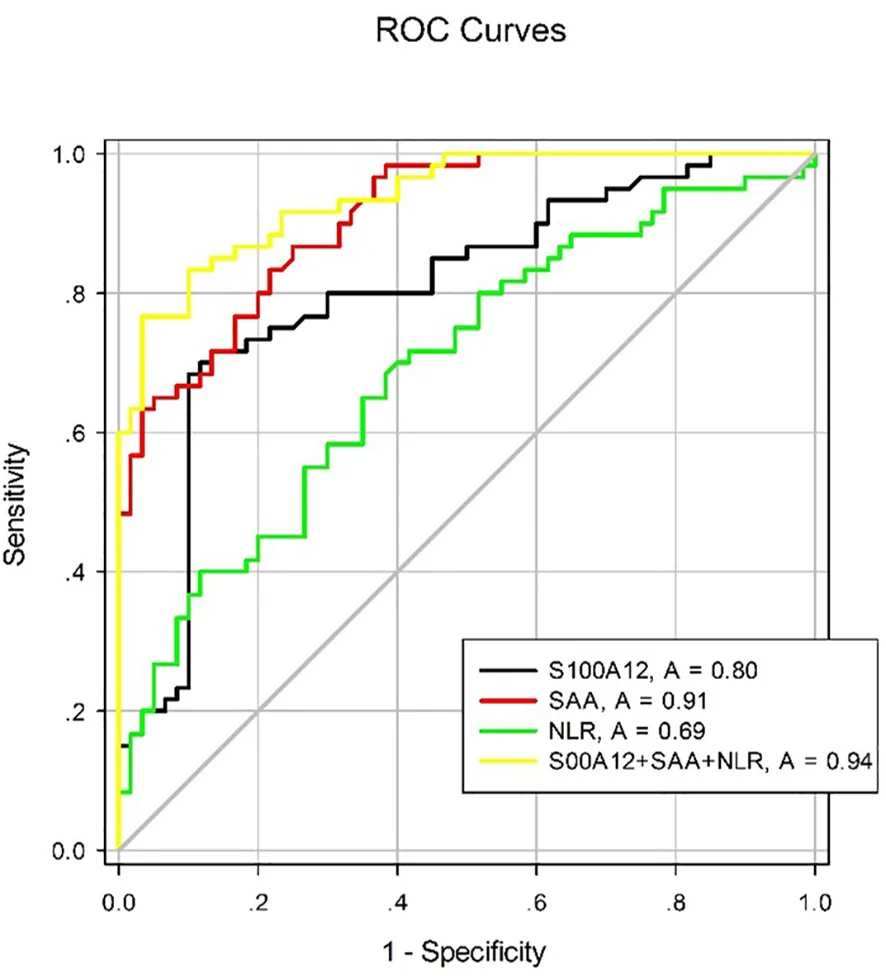
ROC curves of S100A12, SAA, and NLR for predicting acute empyema.

To evaluate the sensitivity and specificity of S100A12, SAA, and NLR for predicting acute empyema, ROC curve analysis was performed for individual biomarkers as well as their combined panel (**Figure 1**; **Table 3**). The combined model of S100A12+SAA+NLR yielded a sensitivity of 76.7% and a specificity of 96.7%, with an AUROC of 0.938 (95% CI: 0.900–0.977, *P* < 0.0001), which was significantly superior to individual S100A12 (AUROC = 0.803, 95% CI: 0.722–0.884, *P* < 0.0001), SAA (AUROC = 0.908, 95% CI: 0.860–0.957, *P* < 0.0001), and NLR (AUROC = 0.694, 95% CI: 0.599–0.788, *P* = 0.0003). These findings confirm that the diagnostic performance of the three-biomarker combination is markedly better than any single parameter, with statistically significant differences (*P* < 0.05).

**Table 3:** ROC curve analysis for the three indicators.

Forecast indicators	Community-acquired pneumonia complicating acute pyothorax					
	AUC	Sensitivity	Specificity	Jordan index	95% CI	P
S100A12 (ng/mL)	0.803	71.7%	86.7%	0.583	0.722–0.884	<0.0001
SAA (mg/L)	0.908	86.7%	75.0%	0.617	0.860–0.957	<0.0001
NLR	0.694	71.7%	58.3%	0.300	0.599–0.788	0.0003
S100A12+SAA+NLR	0.938	76.7%	96.7%	0.733	0.900–0.977	<0.0001

## 4 Discussion

CAP remains a major and prevalent cause of morbidity and mortality worldwide. Severe pulmonary infection or delayed treatment can trigger a variety of pulmonary complications. Previous studies have reported that approximately 20% of CAP patients develop pleural effusion and approximately 30% progress to complicated parapneumonic effusion (CPPE) or empyema (Porcel, 2010). In a cohort of 4,715 CAP patients, 882 (19%) had radiologically confirmed pleural effusion, among whom 261 (30%) were diagnosed with empyema (Falguera et al., 2011). Pleural effusions are generally categorized into three subtypes: uncomplicated effusions, which resolve with antibiotic therapy alone; CPPE, which requires invasive interventions such as chest tube drainage or surgery; and empyema, for which full drainage of pleural fluid is mandatory, as inadequate drainage may eventually progress to pleural fibrosis and impaired lung expansion with disease advancement (Porcel, 2010). Numerous prior investigations have identified risk factors predisposing CAP patients to empyema; timely identification and intervention of these factors help prevent the onset and progression of parapneumonic empyema. Falguera M et al. demonstrated that age <60 years (*P* = 0.012), pleuritic chest pain (*P* = 0.002), and leukocytosis >15,000/mm³ (*P* < 0.001) independently predict acute empyema development (Falguera et al., 2011). In a prospective study via multivariate logistic regression, Chalmers et al. identified several independent predictors for progression of CAP to complicated parapneumonic effusion or empyema: serum albumin <30 g/L, serum sodium <130 mmol/L, platelet count >400 × 10⁹/L, CRP >100 mg/L, history of alcohol abuse (AOR = 4.28, *P* = 0.0006), and intravenous drug use (AOR = 2.82, *P* = 0.03) (Chalmers et al., 2009). As a severe complication of pneumonia, empyema carries a mortality rate up to 20%, accompanied by increased hospitalization expenses and prolonged hospital stay. The development of empyema markedly elevates 30-day mortality and intensive care unit (ICU) admission rates among critically ill patients (Cai et al., 2019).

In recent years, accumulating evidence has indicated that SAA serves as a sensitive biomarker reflecting systemic infection and inflammatory status. Under physiological conditions, circulating SAA concentration is below 10 mg/L. Upon invasion by pathogenic microorganisms including bacteria and viruses, multiple cytokines are released to stimulate hepatocytes to synthesize and secrete abundant SAA, whose concentration rises rapidly within several hours to 10–1,000-fold of baseline values (Zhu et al., 2014; Yan, 2018). SAA is frequently combined with CRP to assist the diagnosis of bacterial infections; previous studies have illustrated that concurrent elevations in SAA and CRP strongly suggest underlying bacterial infection. Benefiting from its relatively short half-life, SAA returns rapidly to normal range once inflammation is effectively controlled (Wang et al., 2020).

Similar to SAA, CRP is another sensitive acute-phase reactant. Its serum concentration increases markedly shortly after the onset of most infectious diseases, starting to rise at approximately 2 h post-infection and peaking within 48 h. CRP increases dramatically in acute bacterial infection, while it shows mild or insignificant elevation during viral infection, rendering it helpful for differentiating bacterial from viral pathogens. Owing to its short half-life, CRP declines rapidly back to baseline within 1–2 days after adequate infection control (Zhu et al., 2012; Wang et al., 2020).

Elevated white blood cell count is one of the most specific laboratory manifestations of acute bacterial infection. Increased neutrophils or prominent left shift on leukocyte differential count provides robust laboratory evidence for empyema secondary to acute bacterial pneumonia (Wang et al., 2020). Patients with empyema are susceptible due to compromised immunity; immune disturbance triggers extensive apoptosis of peripheral lymphocytes. Meanwhile, inflammatory responses such as acute bacterial infection induce elevated total leukocytes, predominantly neutrophils, thereby rendering NLR a feasible biomarker for systemic inflammatory burden. Previous literature has demonstrated that lymphocyte counts correlate closely with disease severity and clinical prognosis (Liu et al., 2014). Accumulating evidence identifies calcium-binding protein S100A12 as a promising inflammatory marker with favorable diagnostic performance in acute bacterial infection, sepsis, and severe infectious diseases (Zhou et al., 2019). According to available clinical data, procalcitonin (PCT) presents unsatisfactory sensitivity and specificity for empyema diagnosis; hence, it was not incorporated into the early diagnostic panel in the present study.

The current study verified that serum S100A12, SAA, and NLR were significantly higher in CAP patients complicated with acute empyema than those without empyema, supporting the three parameters as early predictive biomarkers for empyema development. ROC curve analyses demonstrated that the combined detection of S100A12, SAA, and NLR achieved superior sensitivity, specificity, and the largest area under ROC curve compared with single-index measurement. Accordingly, the combined panel of S100A12+SAA+NLR possesses promising prospects for clinical application in early prediction of acute empyema among CAP patients.

### 4.1 Limitations of the present study

Several limitations exist in this research. Potential confounding factors cannot be excluded: concurrent infections at other sites or underlying autoimmune disorders may cause non-specific elevation of SAA, S100A12, and CRP. Administration of glucocorticoids and antibiotics can suppress inflammatory responses and lead to false-negative results. In addition, case enrollment depended on comprehensive clinical manifestations, which might result in selective inclusion of only severely ill empyema patients and bias intergroup comparison. As a single-center retrospective study with a relatively small sample size, selection bias is inevitable. Further large-sample prospective trials are required to validate our findings.

## Data Availability

The raw data supporting the conclusions of this article will be made available by the authors, without undue reservation.
