# Supplementary material for: Early diagnostic value of serum S100 calcium-binding protein A12, serum amyloid A protein, and neutrophil-to-lymphocyte ratio in patients with acute empyema
Source: Front Cell Infect Microbiol. 2026 Jul 9;16:1740584. doi: 10.3389/fcimb.2026.1740584 (PMC13391829; doi:10.3389/fcimb.2026.1740584)
Supplement: Supplementary file 1 [file Table1.docx]

**Mini Nutritional Assessment Short-Form (MNA-SF)**

| Item |  | Score |
| --- | --- | --- |
| Weight loss in the past 3 months  Appetite over the past 3 months  Mobility  Acute illness in past 3 months  Mental status  BMI（kg/m²） | >3 kg  Unknown  1–3 kg  No weight loss  Severely decreased  Moderately decreased  No change  Bedridden / Wheelchair-bound  Able to get out of bed but unable to go outside  Able to go out freely  Yes  No  Severe dementia /depression  Mild dementia  Normal  <19  19–21  21–23  ≥23 | 0  1  2  3  0  1  2  0  1  2  0  2  0  1  2  0  1  2  3 |

Screening Results (Total score: 14 points)

- 12–14 points: Normal nutritional status
- 8–11 points: At risk of malnutrition
- 0–7 points: Malnutrition
